# Suramin Increased Telomerase Activity in the C6 Glioma/Wistar Experimental Brain Tumor Model

**Published:** 2007-06

**Authors:** Mine Ergüven, Ayhan Bilir, Tuncay Altug, Fadime Aktar, Nuriye Akev

**Affiliations:** 1 *Department of Biochemistry, Istanbul University, Faculty of Pharmacy, 34116 Beyazit, Istanbul-Turkey;*; 2 *Department of Histology and Embryology, Istanbul University, Faculty of Medicine, 34093 Capa, Istanbul-Turkey;*; 3 *Animal Breeding and Research Center, Istanbul University, Cerrahpasa Faculty of Medicine, 34098 Cerrahpasa, Istanbul-Turkey*

**Keywords:** chemosensitivity, C6 glioma, hormesis, suramin, telomerase

## Abstract

Glioblastoma multiforme (GBM) is the most treatment-resistant glioma variant. Significant roles for telomerase in etiology, recurrence and drug resistance of GBM have been highlighted. Suramin (Bayer, Leverkusen, Germany) is an antineoplastic agent that affects many cellular mechanisms including growth factor, purinergic receptor, cytokine and key cellular enzymes signaling. The aim of this study was to investigate whether suramin, 40 mg/kg, i.p., inhibits telomerase activity in a subcutaneous C6 glioma/Wistar experimental brain tumor model using PCR based telomeric repeat amplification assay. In comparison to the control group, suramin increased tumor volume and telomerase activity. We also used transmission electron microscopy to evaluate the alterations of cell morphology. Apoptosis was seen markedly in electron micrographs of the control group and anti-apoptotic activity of telomerase was verified in the electron micrographs of suramin-applied group. The *in vitro* inhibitory effects of suramin on telomerase activity in several cell lines except for brain tumors have been reported. Contrary to *in vitro* reports, our results were the first to demonstrate that suramin increased telomerase activity in a C6 glioma/Wistar experimental brain tumor. Large numbers of drugs exhibited apparent hormetic effects on cultured cancer cells and *in vivo* cancer growth. Several drug examples for their hormetic effects *in vivo* were listed as resveratrol, suramin, and tamoxifen. The action of suramin in the present study could be evaluated as one of the hormetic examples of suramin *in vivo*.

## INTRODUCTION

Telomeres consist of repetitive sequences of G- and C- rich complementary hexanucleotide strands that function as protective structure which cap both ends of the chromosome. Because of the mechanism of conventional DNA polymerases, the replication of DNA molecules can be predicted to result in the gradual shortening of the chromosome by the length of a terminal primer at each cell cycle. With each cell division, the 5’ end of the telomere is shortened by 50-200 nucleotides. After a finite number of replications, telomere reaches a critical length, the senescence checkpoint, and either cell-proliferation arrest or cell death occurs (physiologic aging). If, however, the cell escapes or avoids checkpoint, end-to-end fusion or aberrant recombination of the chromosome occurs, which may contribute to cell death or carcinogenesis (1-4) .

Telomerase is a RNA dependent DNA polymerase that functions as a reverse transcriptase responsible for the de novo synthesis of telomeres. This way telomerese prevents shortening of telomeres. Patients with premature aging syndrome (Werner’s syndrome and Hutchinson-Gilford’s syndrome) have shorter telomeres and loss of telomerase activity as compared to healthy individuals of the same age. Telomerase is practically absent in healthy somatic cells and detected exclusively in immortal cells such as germ cells, stem cells and cancer cells (5). The ability of tumor cells at crisis phase and to bypass cell-cycle checkpoint leads to further shortening of telomeres and the activation of telomerase. The stabilized telomeres provide the ability to proliferate indefinitely (immortalization). About 90% of tumors have active telomerase. All these evidences supports that telomerase seems to play a pivotal role in regulating the structure and function of telomeres during normal physiologic conditions, cell immortalization and tumor progession (6, 7). Telomerase prevents replicative senescence, thus conferring unlimited proliferative potential to cells and was accepted as anti-apoptotic enzyme (8).

Glioblastoma multiforme (GBM) is the most treatment-resistant glioma variant. Significant roles of telomerase in etiology, recurrence and resistance to apoptosis of GBM have been established. Telomerase, therefore, has been proposed to represent a novel and potentially selective target for GBM chemotherapy (9, 10). Rat C6 glioma has been used extensively as an experimental model system for the study of glioblastoma. The rat C6 glioma model is allogeneic in Wistar rats and is not appropriate for evaluating the immune response and survival. Despite this limitation, its use has some merits for the study of the growth, invasion and angiogenesis of glioblastomas. The C6/wistar glioma model resembles GBM characteristics such as necrotic areas, high mitotic index, proliferation and angiogenesis (11).

Suramin is a polyanionic drug that has anti-protozoal, anti-helmintic and anti-neoplastic effects. Suramin has received attention as a potential antineoplastic agent because of its ability to inhibit growth factor-receptor interaction and purinergic receptor-ATP interaction as ATP antagonist, thereby antagonizing tumor cell proliferation and tumor angiogenesis (12-15). *In vitro* telomerase inhibition of suramin in human osteosarcoma cells (MG-63, HOS and SaOS) and murine sarcoma cells (MCG-101) have been established (16, 17). However, any possible modulatory effect of suramin on telomerase activity of brain tumors, yet to be evaluated experimentally. In the present study, we are the first to test the effect of suramin on telomerase activity in C6 glioma/Wistar experimental brain tumors. Contrary to *in vitro* reports, our results demonstrate that suramin increased telomerase activity in rat C6 glioma.

## MATERIALS AND METHODS

### Monolayer Cell Culture

The C6 glioma cell line was obtained from the American Type Culture Collection and maintained in Dulbecco’s Modified Eagle’s Medium and Ham’s F12 media (1:1) (DMEM-F12) containing L-glutamine, Hepes and sodium bicarbonate (Biological Industries, Haemek, Israel). DMEM-F12 was supplemented with % 10 heat-inactivated fetal calf serum (Sigma Chemical Co., St Louis, Missouri), 10.000-units/ml penicilin (Sigma Chemical Co., St Louis, Missouri) and 10 mg/ml streptomycin (Sigma Chemical Co., St Louis, Missouri). The 75 cm^2^ culture flasks (TPP, Trasadingen, Switzerland) were kept in an incubator with a humidified atmosphere of 5% CO_2_ at 37°C and after incubation, medium was discarded. Prior to trypsinazation, the cell layer was washed twice with Ca^+2^- and Mg^+2^ – free phosphate buffered saline (CMF-PBS) (pH7.4). Cells in semi-confluent flasks were harvested using 0.05% trypsin (Sigma Chemical Co., St Louis, Missouri) in CMF-PBS. DMEM-F12 was added for trypsin inactivation. The trypsinized cell suspension was centrifuged and resuspended in DMEM-F12. Cells were counted on a hematocytometer to achieve a concentration of 10^7^ cells in 250 μl of the culture medium (18).

### Animals and Subcutaneous Tumor Implantation

By the approval of the Istanbul University, Institute For Experimental Medical Research (DETAE) Animal Care Investigation Committee, the experiments were performed on *Wistar-Albino* 5-6 week old male rats, weighing 100-150 g, obtained from Animal Breeding and Research Center of Cerrahpasa Faculty of Medicine.

Rats were anesthetized, i.p., with 40 mg/kg pentobarbital. After sterile preparation with betadine and alcohol, 1 × 10^7^ C6 glioma cells in 250 μl of the culture medium were injected subcutaneously into the posterior side of the rat’s neck.

### Experimental Design

Rats were housed in groups of 4 in plastic cages in temperature controlled room with a 12 h light/dark cycle and fed *ad libitum* with commercial feed (Korkut Ilim Yem Sanayi, Antalya, Turkey). After 12-17 day of tumor implantation, tumor volumes were recorded. The tumor volumes were determined in cm^3^ by the W^2^x L/2 formula described by Bullard *et al.* (19). A subcutaneous tumors were achieved to develope in 55% of animals in the present study. The rats in which a subcutaneous tumor development reached a tumor volume of 2 cm^3^ on day 28 were included in our study.

The purpose of our suramin therapy at C6 glioma is not only administering an effective treatment but also to avoid the side-toxic effects. Suramin is highly charged and does not normally cross the blood-brain barrier. In the treatment of brain tumors, high doses of suramin were used to facilitate brain penetration. Recent studies showed that the high dose administration of suramin augmented its efficiency, but side-toxic effects as severe peripheral neuropathy (a dose related axonal neuropathy and severe acute Guillain-Barre-like syndrome) and coagulopathy occurred in human therapy and became significant problem. In an *in vivo* animal model, suramin were administered in various doses ranging from 10 mg/kg to 60 mg/kg and also up to 200 mg/kg and 500 mg/kg. In these studies, low dose 10 mg/kg without recurring administration had no therapeutic effect. At high doses, (60 mg/kg, 200 mg/kg and 500 mg/kg) therapeutic effects were observed with increased polyneuropathy and mortality rates (20-22). Based on these studies, we decided on to start with 40 mg/kg, which was close to high effective dose 60 mg/kg, and which we assumed to be as effective as high dose but with minimize dose-dependent side-effects.

The rats were randomly divided into two groups: Group 1, the control group, received i.p. 0.9% NaCl solution and Group 2, the suramin group, received i.p. 40 mg/kg suramin (Germanin^®^, a kind gift of Bayer, Leverkusen, Germany) twice a week for 45 days. Rats were sacrificed by an overdose of pentobarbital on the 45^th^ day. Tumor diameters and total body weights were recorded. Tumor and brain tissues were dissected. The effects of suramin on C6 glioma tumors were determined by transmission electron microscopy and telomerase activity assay called telomeric repeat amplification protocol assay (TRAP assay). One part of the samples was fixed with 2.5% glutaraldehyde in 0.1 M sodium cacodylate for transmission electron microscopy. The other parts were immediately shock-freezed in aliquots in liquid nitrogen and stored at -80°C for TRAP assay.

### Telomerase Activity-Telomeric Repeat Amplification Protocol (TRAP) Assay

Tissue extracts were prepared and assayed for telomerase activity using a telomerase detection kit (Telo-TAGGG Telomerase PCR ELISA^PLUS^, Roche Diagnostics, Mannheim, Germany), following the manufacturer’s instructions with minor modifications (13, 14). Protein concentration was determined with Bio-Rad protein assay kit (Bio-Rad, Laboratories, Munich, Germany) which is based on Bradford method. An amount of 6 μg of protein extract was added to the reaction buffer including a biotin-labelled P1-TS primer (5’-Biotin-AATCCGTCGAGCAGAGTT-3’) and P2 anchor primer (5’-CCCTTACCCTTACCCTTACCCTAA-3’), telomerase substrate, and Taq polymerase. The 216 bp internal standard which contains the sequence (4620-4779 nt) from bacterial CAT gene flanked by the P1-TS primer and P2 anchor primer was used to identify Taq DNA polymerase inhibitors and false-negative results. After incubation at 94°C for 5 mins, the resulting mixture was subjected to PCR amplification 33 cycles of 30s at 94°C, 30s at 50°C, and 90s at 72°C. Reaction products were separated on 12% non-denaturing polyacrylamide gels and stained using Silver Staining Kit (Bio-Rad Laboratories, Munich, Germany) to test the specificity of PCR results. 5 μl of the amplified PCR product was denatured and hybridized to two different digoxigenin (DIG)-labeled detection probes:

- P3-T was complementary to telomeric repeat sequences and used for specific detection of telomerase-mediated amplification;
- P3-IS complementary to the internal standard and used for specific detection of amplified Internal standard (IS). The mixture was immobilized by biotin labeled TS primer to a streptavidin coated microtiter plate, and was detected with an anti-digoxigenin antibody conjugated to peroxidase (Anti-DIG-POD) which was involving the IgG subclass. Absorbance values were measured using a microtiter plate reader (Thermo Multiskan EX, Milford, USA) at 450 nm with reference wavelength of 690 nm. Telomerase activity was calculated using the formula: ΔA= Δ_450_-Δ_690_ (Δ_450_: Absorbance value at 450 nm, Δ_690_: Absorbance value at 690 nm). All assays were performed in triplicate.

### Cell Morphology-Transmission Electron Microscopy (TEM)

Harvested spheroids were fixed with 2.5% glutaraldehyde in 0.1 M sodium cacodylate buffer and post-fixed in 1% osmium tetraoxide in 0.1 M sodium cacodylate buffer for 1 h at 4°C. Cells were incubated in 1% uranyl acetate for 1 hour at 4°C, dehydrated in graded acetone series and embedded in Epon 812. Samples were cut on a microtome (Leica MR 2145, Heerbrugg, Switzerland) and 70 nm thick sections were mounted on copper grids. They were stained with 5% uranyl acetate and counterstained with Reynold’s lead citrate. Sections were examined with a Jeol-Jem 1011 transmission electron microscope.

### Statistical Analysis

The results were evaluated by using the Statistical Software SigmaStat Version 12.0 (Version 12.0; SPSS, Inc.,Chicago, IL). Results are presented as mean ± SEM. Independent Student *t*-test was used for the comparison of tumor volume and telomerase activity between the control group and the suramin group. p_t-test_ values, which are smaller than 0.05, were considered statistically significant. The correlation between these two parameters was determined via 2-tailed Pearson correlation test and *p*<0.01 were considered statistically significant.

## RESULTS

### Tumor Volume

**Figure 1:**
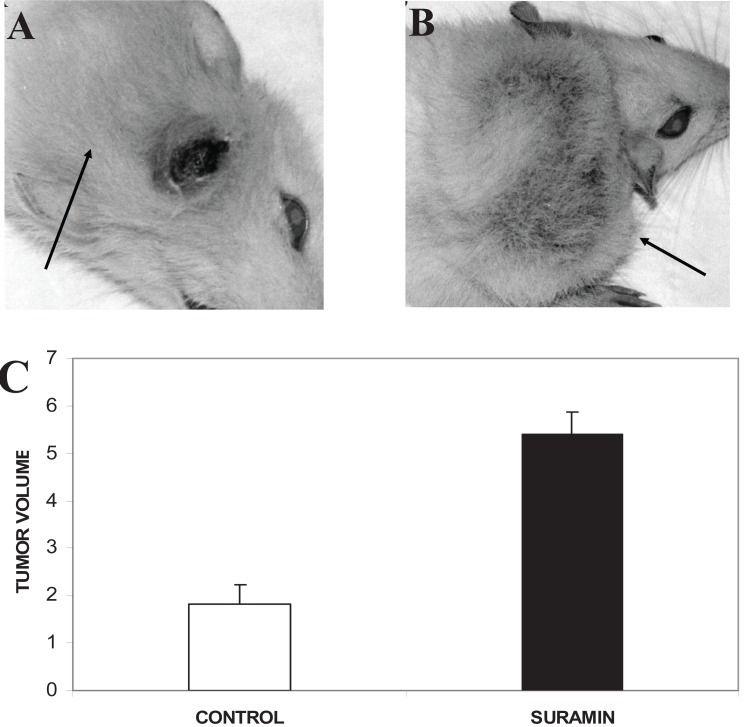
Tumor photographs of sacrificed rats after high dose pentobarbital administration and the figure about the alterations of tumor volume. Rats received, i.p., 40 mg/kg suramin twice a week. Arrows show the tumors originated from C6 glioma cells in subcutaneous area of neck. (A) The rat from control group with tumor volume: 5.26 cm^3^; (B) 40 mg/kg suramin treated rat which had the highest tumor volume: 8.15 cm^3^; (C) Effect of suramin on mean tumor volume. Suramin increased tumor telomerase activity. Mean tumor volume in cm^3^ (*p*<0.05).

The mean tumor volume in the control group was 1.821 ± 0.396 cm^3^ (Fig. 1A, 1C) and 5.387 ± 0.495 cm^3^ in the suramin group (Fig. 1B, 1C). The difference in tumor volume between the control group and the suramin group for these parameters was significant at 0.05 level (p:0.03, *p*<0.05). In the control group, we noticed that the tumors, in a substantial number of rats, regressed spontaneously in volume (17/20) which was started on day 28. Suramin as a chemotherapeutic agent, interestingly, increased tumor volume. As rats with regressed tumor displayed severe cancer cachexia, we decided that they could not survive throughout the entire intended study period. Consequently, all rats including the suramin group was sacrificed on day 45.

### Telomerase Activity

**Figure 2:**
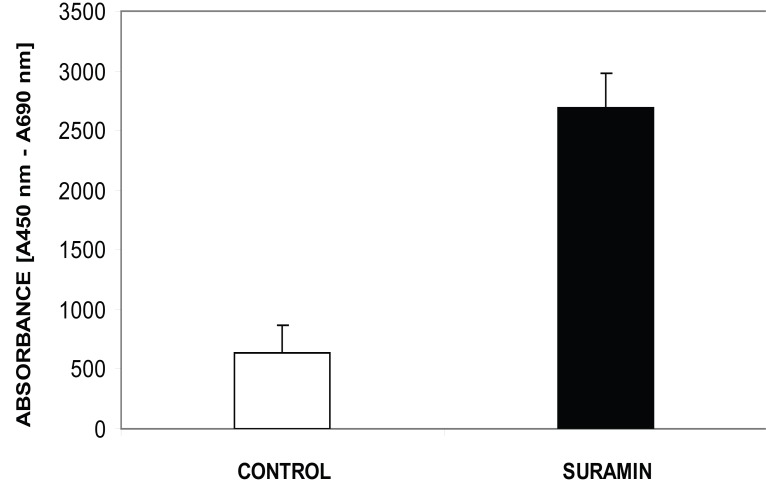
The effect of suramin on telomerase activity. Suramin increased the telomerase activity of glioma. Mean telomerase activity, ΔA= ΔA450-ΔA690 (*p*<0.05). All assays were performed in triplicate.

The mean telomerase activity (ΔA_mean_) in the control group was 0.674 ± 0,229 absorbance units. Low telomerase activity in the control group was concomitant with a reduction of tumor volume. The mean telomerase activity (ΔA_mean_) in the suramin group was 2.686 ± 0.293 absorbance units (Fig. 2). Suramin increased telomerase activity as well as tumor volume. The difference in telomerase activity between the control group and the suramin group was significant at 0.05 level (p:0.02, *p*<0.05). The correlation between tumor volume and telomerase activity for the control and suramin group was significant at the 0.01 level (p:0.0095, *p*<0.01). This correlation supports the connection between the proliferative capacity and high telomerase activity (Fig. 1B).

Recent studies have shown that, unlike humans, almost all somatic tissues of mice and rats have detectable telomerase activity (25). Yamaguchi Y *et al*. investigated telomerase activity in fetus, neonate, 4 week (adolescent), 7 week (adult) and 2 years old rats. They showed that various rat organs showed three chronological patterns with respect to the expression of telomerase activity. In brain the high telomerase activity presented at embryo, remained high at neonate, then diminished rapidly, and was almost undetectable in adults. Therefore, this group resembles normal human tissues in which telomerase activity diminishes as cells are differentiated (26). Sakitani et al induced oligo-astrocytomas by using N-ethyl-N-nitrosourea (ENU) and showed that telomerase activity of oligoastrocytomas, including early neoplastic lesions were increased as compared to normal brain (27). In the present study the 7-weeks old rats and the glial cell strain, C6, which was cloned from a rat glial tumor induced by N-nitrosomethylurea by ATCC after a series of alternate culture and animal passages were used. To define telomerase activity, the brains taken from control group which were inoculated with C6 glioma but no chemotherapy applied, and healthy rats which were not inoculated with C6 glioma cells and not treated with suramin were used as controls in addition to lysis buffer. Consistent with previous results, brains possessed telomerase activities in only trace amounts.

### Cell Morphology

**Figure 3:**
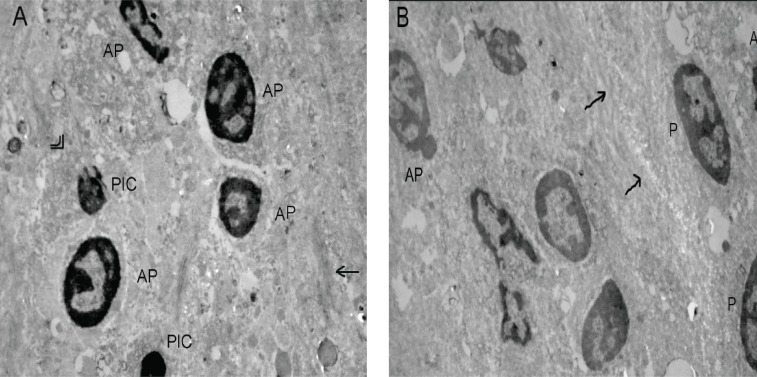
Transmission electron micrographs of C6 glioma tumor sections from rats in the control group and the suramin group. Cells with apoptotic nucleus in the control group and cells with proapoptotic nucleus in the suramin treated group were the distinct ultrastructural differences were observed in electron micrographs. (A) The control group (x 5000, original magnification). AP: Cells with apoptotic like nucleus, PIC: Cells with pinocytotic nucleus, Arrow (→): Membrane fusion, Double arrows: Cytoplasm fusion with lytic changes; (B) The suramin group (x 6000, original magnification). AP: Cells with apoptotic like nucleus, P: Cells with proapoptotic like nucleus, Double arrows (→): Membrane fusion.

Contrary to the control group of C6 glioma cells *in vitro* (data not shown), cells with apoptotic like nucleus which supported the occurrence of spontaneous regression were observed in tumor sections of the control group (Fig. 3A). The existence of C6 glioma cells mostly with proapoptotic nucleus were observed in tumor sections from the suramin group and this evaluation strengthen the evidence of anti-apoptotic effect of suramin led to increase in tumor volume and telomerase activity (Fig. 3B). Cell membrane fusions and cells with pinocytotic nucleus were the common morphological structures observed in two groups. Cytoplasm fusion with lytic changes in the control group was also noteworthy. The membranous inclusion bodies as an indicator of glycosaminoglycans accumulation that was demonstrated in glioma and endothelial cells in the tumor as well as glioma cells by Takano *et al*. (28) were not detected in the suramin group in the present study.

The 40 mg/kg suramin administration was chosen to reach therapeutic efficiency with minimal side-effects, however, this dose revealed no therapeutic response to subcutaneous C6 glioma/Wistar model.

## DISCUSSION

The glioblastoma multiforme (GBM) is the most malignant glioma variant with approximately 9 to 12 months of median survival, after diagnosis (29). Rat C6 glioma is a common experimental model system for the study of glioblastoma growth and invasion. The C6/Wistar model has been used extensively, but immune response due to the allogenicity is the main limitation. Beutler *et al*. described the presence of allogeneic MHC proteins in implanted tumors and showed that they could be up-regulated resembling a therapeutic response which was not normally seen in syngeneic models (30). Parsa *et al*. reported a strong immunological reaction to the C6-tumor implanted both intracranially and in the flank of the Wistar model (31). Similar results were found for the allogenicity of the C6/Wistar rat glioma model in the present study. The first evidence is a subcutaneous tumor development was almost never observed in 100% of animals in previous studies. Such as a study done by Arrieta O. *et al*. (32), they have developed C6 glioma subcutaneous tumors only in 80% of rats and a subcutaneous tumor developed in 55% of animals in our study. In contrast to increased growth of tumor volume in the suramin group, spontaneous regression characterized by decreasing tumor volume was observed in the control group. The inhibitory effects of suramin on cytokines such as TNF-α which has role in either in apoptosis or anti-apoptotic activity, interleukin-2, and interferon-γ were shown (33-36). As an example for the tumor volume were 5.26 cm^3^ and 8.15 cm^3^ for the control group and the suramin group, respectively (Figure 1). All these results could be interpreted as the consequences immunosuppression inhibition.

Suramin is an antineoplastic agent that affects many cellular mechanisms including growth factor, purinergic receptor, cytokine and key cellular enzymes (i.e. protein kinase C etc.) signaling. Takono *et al*. reported the inhibitory effect of suramin on C6 glioma cell proliferation *in vitro* and in the brain (28). Arrieta *et al*. showed the inhibitory effect of suramin on the tumor growth of the C6 glioma cells which were implanted subcutaneously into the left thigh of Wistar rats (32). The inhibitory effect of suramin on telomerase activity in several cell lines such as human osteosarcoma cells (MG-63, HOS and SaOS) and murine sarcoma cells (MCG-101) except for brain tumors *in vitro* have been reported (16, 17). In the present study, suramin behaved like a switch from apoptosis to carcinogenesis through increasing tumor growth and telomerase activity contradicting with their results.

Eichhorst *et al*. showed that suramin inhibits death receptor-induced apoptosis in hepatoma and lymphoma cells *in vitro* and fulminant apoptotic liver damage in mice. They also mentioned that the antiapoptotic mechanism is specific to cell type and is caused by reduced activation, but not altered composition, of the death-inducing signaling complex (DISC), and by inhibition of the initiator caspases 8, 9 and 10. In the same study they also mentioned that suramin also shows similar effects in *in vivo* models; apoptotic liver damage induced by CD95 stimulation and endotoxic shock mediated by tumor-necrosis factor (TNF) are inhibited in mice (33). Besides its antiapoptotic property, several studies showed the immunosupressive effect of suramin via cytokine inhibition such as TNF-alpha, IL-2, IL-6 and interferon (33-36). In our study, parallel to these findings suramin could increase tumor growth and telomerase activity via the inhibition of immune system and apoptosis. Cancer cachexia and cells with apoptotic nucleus observed in the control group, and decreased cancer cachexia and substantial number of cell with proapoptotic nucleus in the suramin group were all together supported the inhibition of immune response and apoptosis by suramin. The immunosupressive effect of suramin could abolish limitations of allogenicity and increase tumor take in the present study.

The effects suramin on telomerase activity were rarely investigated and limited with osteosarcoma and murine sarcoma cell lines as we mentioned before (16, 17). The molecular mechanism of suramin inhibitory effect on telomerase activity remains to be defined in these studies. The researchers speculated that suramin exerted this effect mainly through direct intracellular effect on telomerase activity via its ability to inhibit reverse transcriptase and DNA polymerase; and also through inhibition of telomerase stimulators (16, 17, 37). Previous studies clarified the activators and the inhibitors of telomerase in various cancer types. The activators of telomerase are c-myc and Sp1 (38), nuclear factor κβ (nf-κβ) (39), TNF-α (40), protein kinase C alpha (PKCα) and protein kinase B (PKB/Akt) (41, 42), E2-F (43), epidermal growth factor (44), hypoxia-inducible factor 1 α (HIF1- α) via mitogen activated protein kinase (MAPK) signaling pathway (45, 46), methionine aminopeptidase-2 and bcl-2 (37). The inhibitors are tumor growth factor beta (TGF β), Wilm’s tumor 1 (WT-1), p53, Mad 1 and Rb (47), interferon (48). In the present study, suramin might have increased telomerase activity by preventing the effects of telomerase inhibitors on telomerase activators and/or stimulating telomerase activators. For example, the growth factor signaling pathway plays important role on tumor growth and on telomerase activation. Although suramin exerts its antineoplastic effect by inhibiting growth factor and receptor interaction, recent studies on variety of cancer cells revealed stimulatory effect of suramin on growth factor receptors (49). This effect of suramin could be an another mechanism that contributed to tumor growth and telomerase activation.

The stem cells with high telomerase activity in tumors are thought to be the origins of tumor occurrences, recurrence and drug resistance in GBM. Studies showed that the rat C6 glioma contained 0.4% subpopulation of cancer stem cells *in vitro*. Although we did not isolate and characterize stem cell subpopulations in the C6 glioma brain tumor model, recent studies shed light on the existence of heterogeneous populations including stem cells and non-stem cells in tumors (50-52). It was shown that suramin had no or lower effect on unstimulated cells (16, 35). As stem cells are quiescent cells, it could be interpreted as suramin might not show its inhibitory effect. Telomerase activity coming from this stem population is presumably involved in total telomerase activity obtained from tumor extracts. This could be the one of the reason for pleiotropic effect of suramin effect which was contributed to tumor growth.

The hormesis theory definition proposes the low-dose beneficial and high-dose detrimental pattern of several drugs for carcinogenicity. Large numbers of drugs exhibited apparent hormetic effects on cultured human cancer cells, causing stimulation of cell growth or related changes at low doses, followed by inhibition at higher doses. This condition was also the same for *in vivo* cancer growth. Several drug examples for their hormetic effects *in vivo* can be listed as resveratrol, suramin, and tamoxifen. Comparison of the dose and concentration ranges commences that hormesis could be an issue *in vivo* (53). We think that our study could be one of the hormetic examples of suramin *in vivo*.
